# A critical period of progesterone withdrawal precedes menstruation in macaques

**DOI:** 10.1186/1477-7827-4-S1-S6

**Published:** 2006-10-09

**Authors:** Ov D Slayden, Robert M Brenner

**Affiliations:** 1 Division of Reproductive Sciences, Oregon National Primate Research Center, Oregon Health & Science University, Beaverton, Oregon, USA

## Abstract

Macaques are menstruating nonhuman primates that provide important animal models for studies of hormonal regulation in the uterus. In women and macaques the decline of progesterone (P) at the end of the cycle triggers endometrial expression of a variety of matrix metalloproteinase (MMP) enzymes that participate in tissue breakdown and menstrual sloughing. To determine the minimal duration of P withdrawal required to induce menses, we assessed the effects of adding P back at various time points after P withdrawal on both frank bleeding patterns and endometrial MMP expression. Artificial menstrual cycles were induced by treating the animals sequentially with implants releasing estradiol (E_2_) and progesterone (P). To assess bleeding patterns, P implants were removed at the end of a cycle and then added back at 12, 24, 30, 36, 40, 48, 60, or 72 hours (h) after the initial P withdrawal. Observational analysis of frank bleeding patterns showed that P replacement at 12 and 24 h blocked menses, replacement at 36 h reduced menses but replacement after 36 h failed to block menses. These data indicate that in macaques, a critical period of P withdrawal exists and lasts approximately 36 h. In other similarly cycled animals, we withdrew P and then added P back either during (12–24 h) or after (48 h) the critical period, removed the uterus 24 h after P add back and evaluated endometrial MMP expression. Immunocytochemistry showed that replacement of P during the critical period suppressed MMP-1, -2 and -3 expression along with menses, but replacement of P at 48 h, which failed to suppress mense, suppressed MMP-1 and MMP-3 but did not block MMP-2. We concluded that upregulation of MMPs is essential to menses induction, but that after the critical period, menses will occur even if some MMPs are experimentally blocked.

## Review

Macaques are excellent models for the study of menstruation because, like women, they display 28-day menstrual cycles [1-3]. The macaque uterus, which is anatomically similar to the human uterus, has been morphologically characterized into well defined functional zones [4,5]. In this classification, the surface epithelium and an underlying band of stromal cells was defined as Zone I. Zone II, slightly deeper, contains glands that run perpendicular to the surface. Deeper still, Zone III contains glands that are branched, and the deepest zone, Zone IV is the basal layer adjacent to the myometrium, where the glands terminate. Secretory differentiation occurs in Zone I-III, and these zones are frequently termed the endometrial functionalis; this is the zone that sloughs during menses. Artificial menstrual cycles can be induced in macaques by sequentially treating the animals with estradiol (E_2_) and progesterone (P) [6,7]. In this model, treatment with E_2 _for 2 weeks induces an artificial proliferative phase and treatment for the next 2 weeks with E_2 _plus P induces an artificial secretory phase. Withdrawal of P at the end of the secretory phase completes the cycle and stimulates menstruation. We refer to the 3–4 day period after P withdrawal as the luteal-follicular transition (LFT) [8,9].

In women and nonhuman primates the decline of P is followed by a cascade of endometrial events including increased expression of matrix metalloproteinase (MMP) enzymes [7,10]. The MMPs are capable of degrading the extracellular matrix and play a role in the dissociation of tissues in the upper endometrial zones (Zone I-III) during menstruation [10-12]. Several studies report that P specifically inhibits MMP expression in human endometrial stromal cells in vivo and tissue explants in vitro [13-15]. Expression of MMP-1, -2, -3, -10, -11, and -14 in the rhesus macaque endometrium peaks on days 2–4 of the LFT, then declines spontaneously after menstruation during the proliferative phase of the cycle even though P is absent. Paradoxically, progesterone receptor (PR) consensus sequences have not been identified on the genes for these menstruation-associated MMPs. Therefore, P mediated regulation of endometrial MMPs appears to be indirect. Because of the focal nature of menstrual breakdown and the restriction of MMP expression to the mid and upper functionalis zones, it has been suggested that local factors such as specific cytokines mediate the effects of P withdrawal on the endometrium. For instance, TGF-beta has been identified as a mediator of MMP-7 suppression by P [16]. Also, endometrial bleeding associated factor (ebaf; lefty -A) has been identified as an inhibitor of TGF-beta B [17], and lefty-A can induce expression of several MMPs in human endometrial stromal cells. Because P can still suppress MMPs upregulated by lefty-A in vitro [18], the full mechanism through which P regulates endometrial MMPs remains to be clarified.

Kelly et al [19] have suggested that regulation of menstruation occurs in two phases. In the first phase, vasoconstriction and local cytokine expression are initiated by P withdrawal; this reaction is mediated through PR in the perivascular cells surrounding spiral arteries. Subsequently, various lytic events are initiated by leucocytes and other cells that lack PR. This hypothesis implies that the first, but not the second phase could be blocked by the readministration of P. In this view, P withdrawal is followed by a "critical period" after which, tissue breakdown, sloughing and bleeding become inevitable. Here we review these issues and expand on studies we previously reported [20] in which we added back P at various times after P withdrawal to assess the length of the critical period in macaques. Our results confirmed the view that there is a physiologically significant critical period underlying the menstrual process.

### Experimental Design

**Figure 1:**
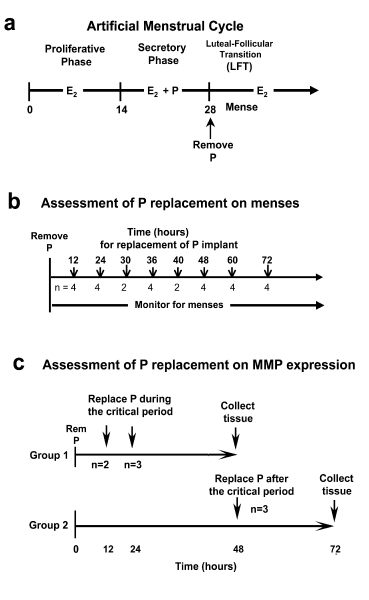
**(A) **Experimental protocols. The artificial menstrual cycle. Sequential treatment with E_2 _alone (Proliferative Phase) and then E_2 _+ P (secretory phase) primes the endometrium for menstruation during the Luteal Follicular Transition (LFT) after P implant removal. **(B) **Assessment of P add back on menses. P implants were removed at the end of the cycle. P implants were then replaced in the animals at 12, 24, 30 36, 40, 48, 60, or 72 hours (h) after initial P withdrawal. Control animals (n = 1/time point) received blank Silastic implants. **(C) **Assessment of P add back on MMPs. P was withdrawn as described above, and then either replaced at 12 and 24 hours during the critical period (Group 1), or at 48 hours after the critical period (Group 2). Tissues were collected at 48 h for group 1 and 72 hours for group 2. Control animals did not have P implants replaces and tissues were collected at either 48 or 72 h after P withdrawal.

**Figure 2:**
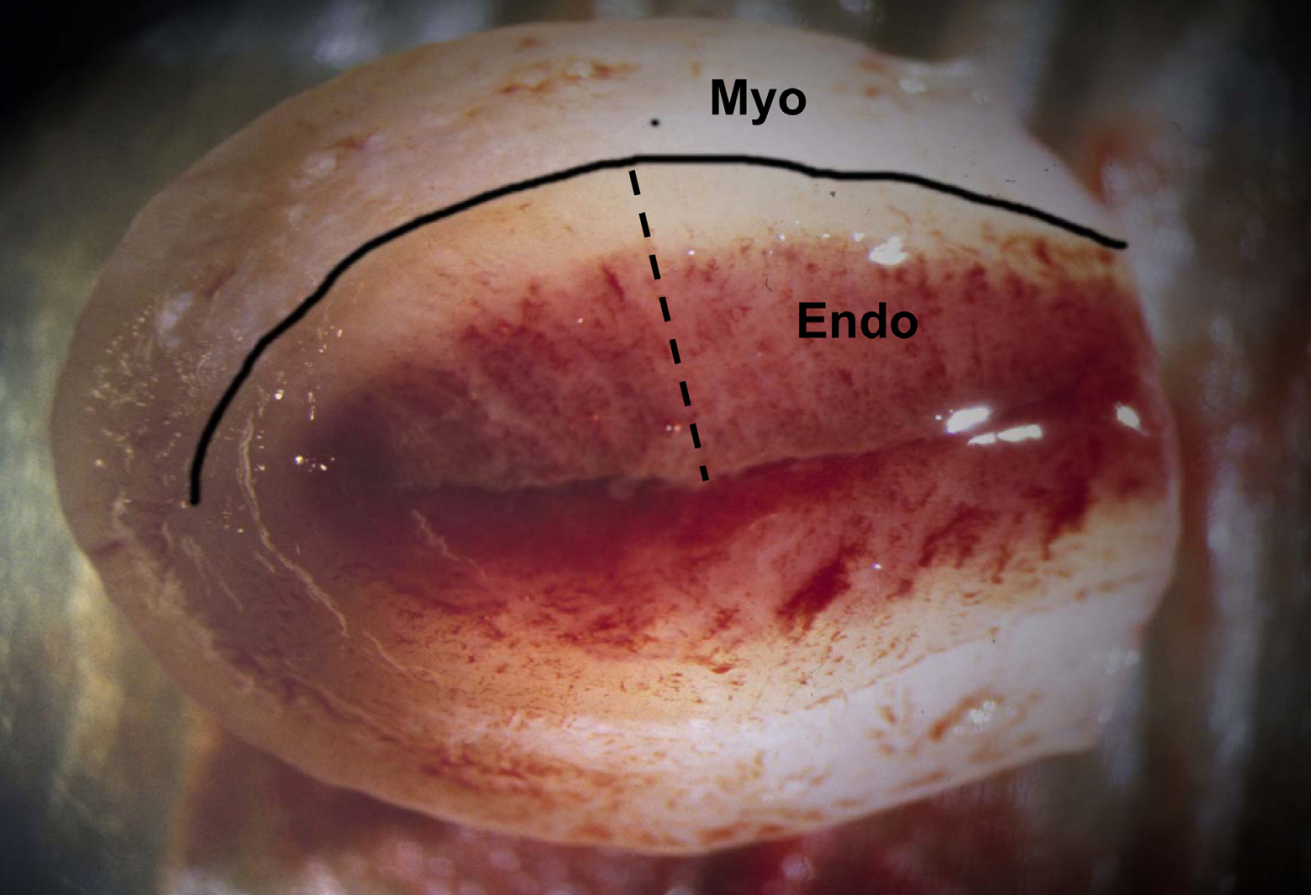
A rhesus macaque uterus from an early menstruating animal 48 hours after P withdrawal. For this photograph the uterus has been separated from the cervix and cut in half along the longitudinal axis. A black line has been drawn to delineate the endometrial-myometrial border. The beginning of tissue bleeding is evident in the functionalis zone. The black dashed line shows the full-thickness plane for histological sectioning. Endo = endometrium; Myo = myometrium.

The veterinary staff of the Oregon National Primate Research Center (ONPRC) provided animal care during this work and all studies were approved by the ONPRC Animal Care and Use Committee. Sexually mature rhesus (*Macaca mulatta*) and pigtailed macaques (*Macaca nemestrina*) were ovariectomized and treated to induce artificial menstrual cycles (Fig. 1a) as previously described [6]. To compare the timing and duration of menses between rhesus and pigtailed macaques, 12 of each species were checked for menses daily for 2 artificial cycles. To obtain this record, the perineum was inspected visually and if blood was observed, this was recorded as frank bleeding. If no blood was evident, the vagina was swabbed with a cotton-tipped swab and blood on the swab was recorded as spotting. Minimal differences were evident between these species, and all further experimental work was conducted in *Macaca nemestrina*. To test the effect of P replacement on menses during the LFT, artificial menstrual cycles were induced in pigtailed macaques and after one cycle, the P implants were removed (leaving E2 in place) and either a blank or a P implant was replaced at 12, 24, 30 36, 40, 48, 60, or 72 hours (h) after initial P withdrawal. Control animals (n = 1/time point) received blank Silastic implants. Fig. 1b depicts this experimental design. In a later experiment, P was withdrawn (time 0) and then replaced at either 12 h, 24 h or 48 h later and uteri were collected 24 hours after P add-back as shown in Fig. 1c. Uterine samples were sectioned and analyzed by immunocytochemistry for expression of MMPs. In Fig. 2, a black dashed line has been drawn to illustrate the plane of the histological sections depicted in subsequent photomicrographs. Histology and immunocytochemistry for MMP-1, MMP-2 and MMP-2 was conducted on microwave-stabilized cryosections as previously reported [7,21].

### Effects of P withdrawal on menstruation in rhesus and pigtailed macaques

**Figure 3:**
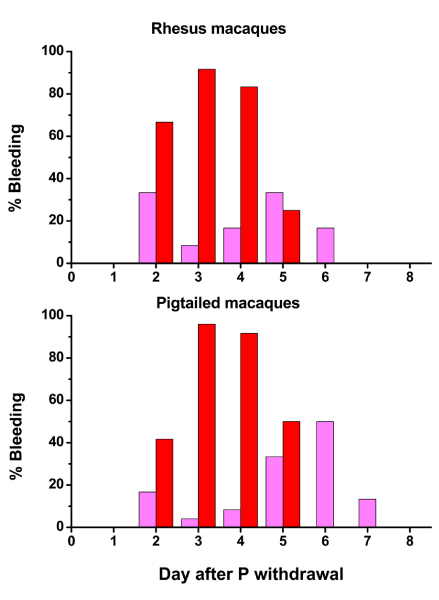
Effects of P withdrawal on menstrual bleeding in rhesus and pigtailed macaques. In this chart, each bar represents the percentage of animals (n = 12) that were bleeding on each day of the LFT. Pink bars depicts swab-positive spotting, and red indicates frank menses.

Figure 3 shows the effect of P withdrawal on menstrual bleeding in 12 rhesus and 12 pigtailed macaques monitored for 2 artificial menstrual cycles. Menses began in most of the animals on day 2 of the LFT, and all of the animals menstruated frankly for 2–3 days. Onset of menses appeared to be slightly more rapid for rhesus macaques with 64% menstruating frankly on Day 2 compared to 40% for pigtailed monkeys. Similarly, more pigtailed macaques were swab positive on day 6 and 7.

**Figure 4:**
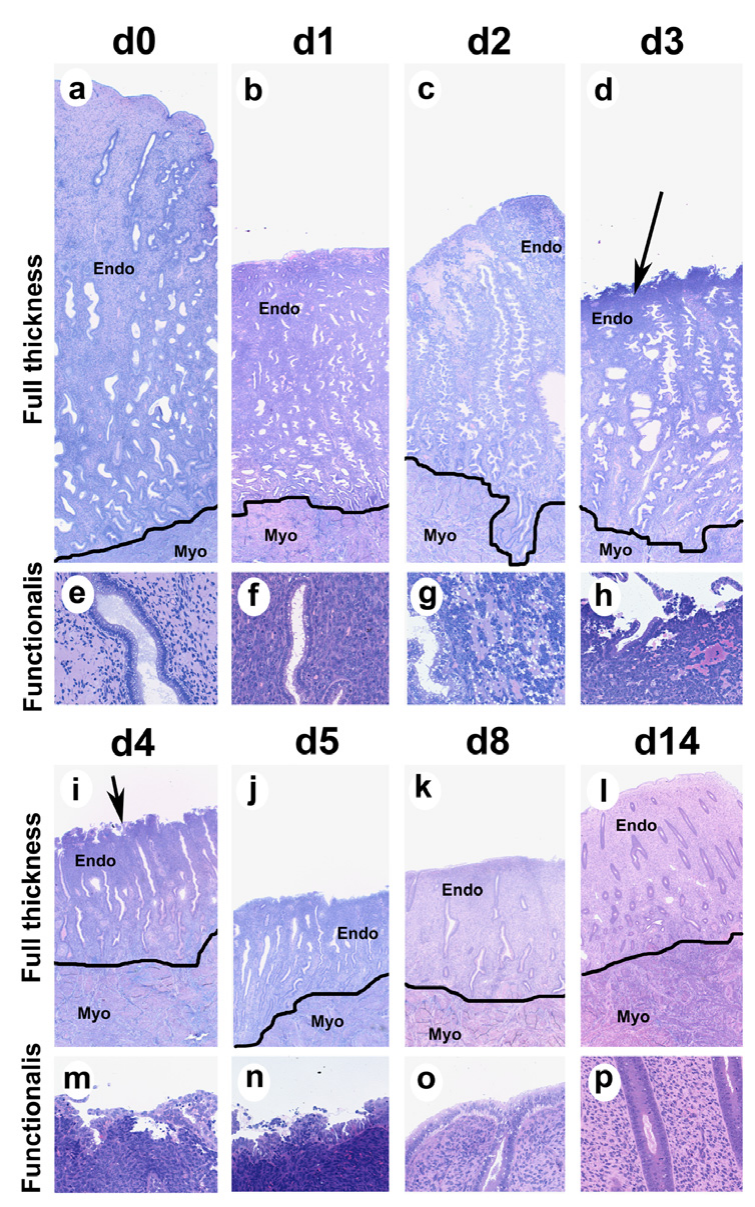
Endometrial histology in the pigtailed macaque. Photomicrographs of GMA sections showing endometrial histology from pigtailed macaques on day 0,1, 2, 3, 4, 5, 8, and 14 of the artificial menstrual cycle. a-d and i-l show full-thickness sections all at the same magnification (25× original magnification). A line has been drawn to delineate the endometrial-myometrial border. High power photographs of the upper functionalis zone are shown in e-h and m-p (200× original magnification). At the end of the cycle (a, e) the endometrium is thick and the glands are sacculated due to the effect of P. P withdrawal induced endometrial shrinkage (day 1) and the beginning of bleeding by day 2 (note tissue breakdown in (g). The sloughing menstrual surface is clearly evident on d3 and d4 (Arrows in d and i point out menstrual surface). By day 5 the luminal surface is recovered with epithelial cells. During the proliferative phase (d8 and d 14) the endometrium regrows with tubular glands that show abundant mitotic cells (p). Endo = endometrium; Myo = myometrium.

The cyclic changes in the endometrium in the pigtailed macaque are depicted in Fig. 4, which shows histological sections of pigtailed macaque endometrium collected from animals on days 0(28), 1, 2, 3, 4, 5, 8, and 14 of the artificial menstrual cycle. Day 0 is the end of the secretory phase, before P declines, days 1–5 correspond to the LFT (after P declines) and days 8 and 14 correspond to the proliferative phase of the cycle. On day 0, the secretory endometrium is thick, the glands are notably sacculated, and there is extensive stromal expansion due to P-depended secretion of extracellular matrix in Zones I and III of the endometrium. On day 1 of the LFT, P withdrawal results in shrinkage of the endometrium and a notable compaction of the stromal cells in Zones I and II of the endometrial functionalis. Endometrial tissue fragmentation in the mid and upper functionalis zone is clearly evident on days 2 and 3, and by day 4 most of the upper 1/2 of the endometrium has sloughed. The lower functionalis and basalis showed no signs of fragmentation but from days 2–6, the glandular epithelial cells in the basalis underwent extensive apoptotic cell death similar to the rhesus macaque [22]. Around day 5 mitotic activity is evident in the uppermost regions of the surviving glands, and by day 8 the endometrial lumen is fully healed and regrowing through both stromal and glandular proliferation. At the end of the proliferative phase (day 14) the endometrium has regrown but remains non secretory with tubular proliferative glands.

**Figure 5:**
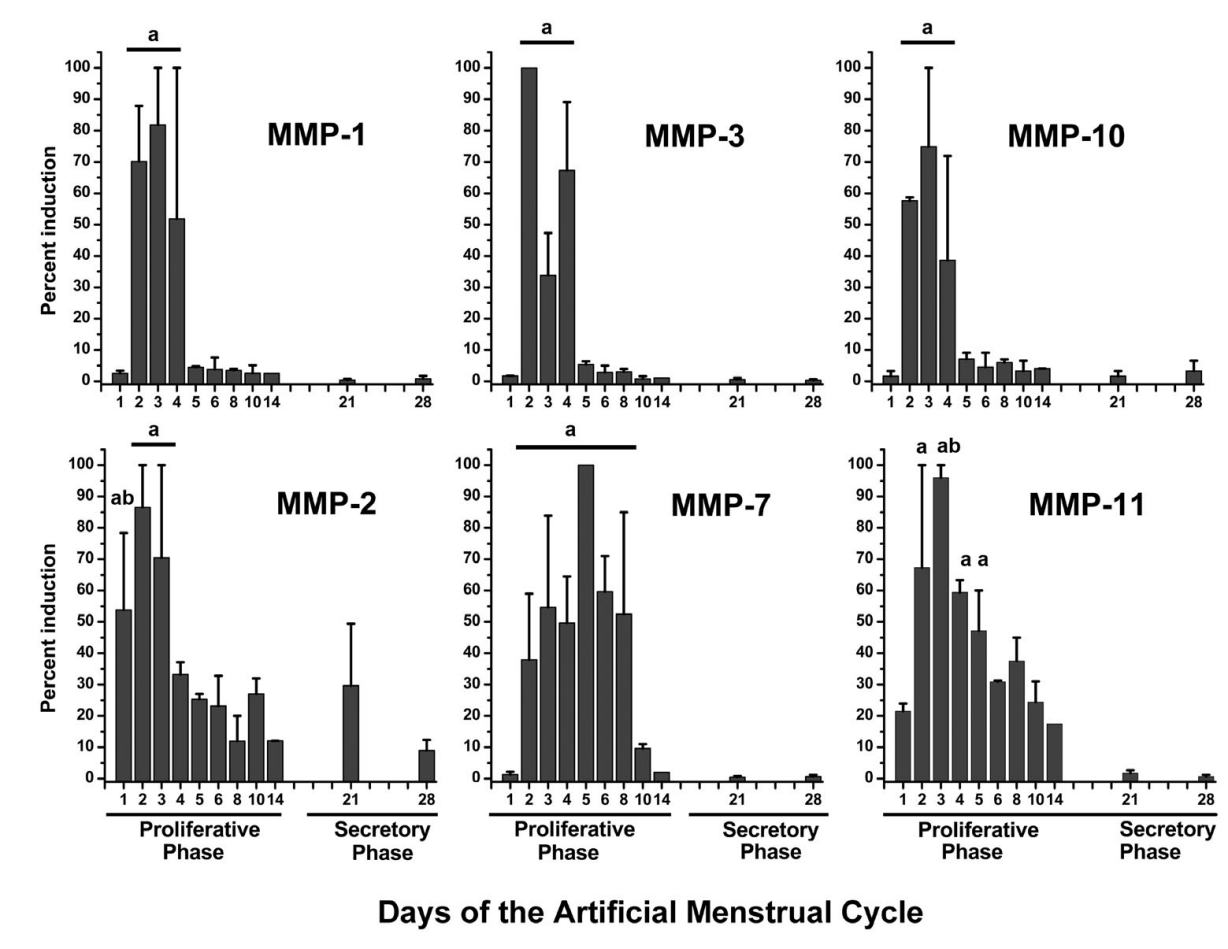
Expression of MMP transcripts during the menstrual cycle in rhesus macaques. MMP expression was assayed by Northern Hybridization and expressed as a percent of the maximum for the group. Values were analyzed previously [6,7] by ANOVA followed by Fisher's Protected LSD. In each case MMPs were maximal during the menstrual period at the beginning of the proliferative phase. Bars with similar subscripts were significantly different (P < 0.05) from those without subsubscripts.

**Figure 6:**
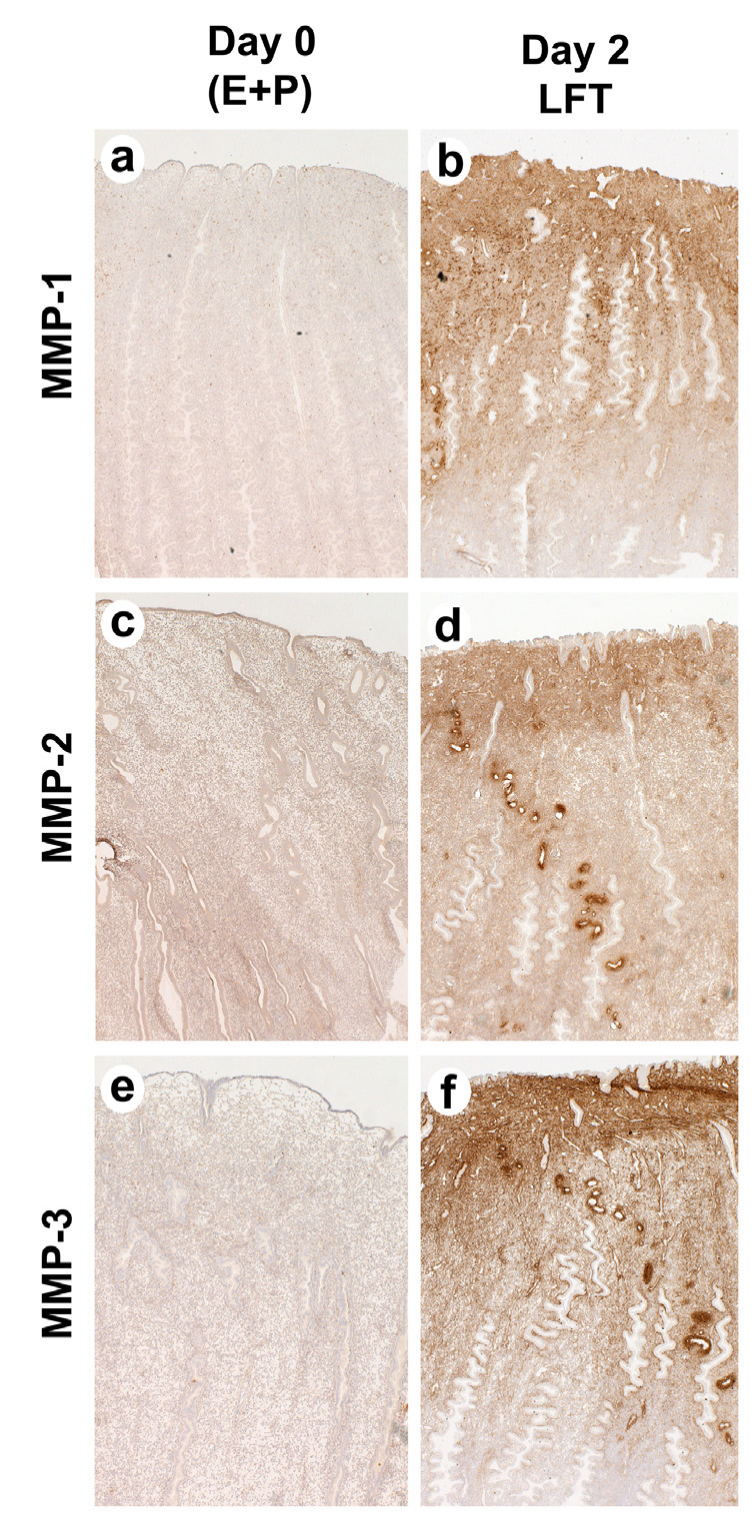
Immunostaining for MMP-1, MMP-2 and MMP-3 in the pigtailed macaque endometrium. Samples were collected either on day 0 (a,c,d) or on day 2, 48 hours after P withdrawal (b,d,f). Note the strong gradient of MMP immunostaining with the strongest signal in the upper and mid functionalis zone. Magnifications (50× original magnification).

In earlier studies [6,7] we reported the pattern of expression of various MMP mRNAs during the menstrual cycle of rhesus macaques (Fig. 5), and we noted that these MMPs were localized in the uppermost endometrial zones. Similarly, in the pigtailed macaque, maximal expression of MMPs was observed during the LFT. Figure 6 shows immunostaining for MMP-1, MMP-2 and MMP-3 in the pigtailed macaque endometrium 48 hours after P withdrawal. There is a marked gradient of MMP staining with the strongest signal in the upper and mid functionalis zones.

### Effects of P replacement on menstruation

**Figure 7:**
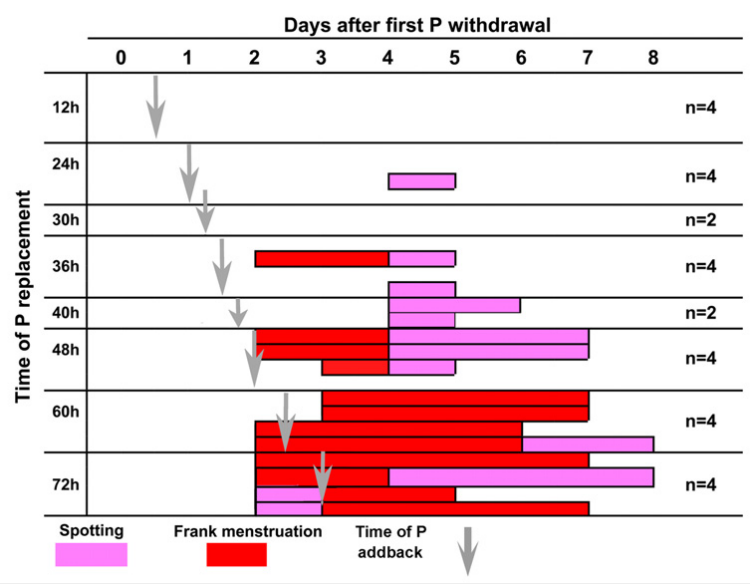
Effect of P replacement during the LFT. This chart shows menstrual bleeding on each day after P withdrawal during the luteal-follicular transition. P implants were replaced at 12, 24, 30, 36 40 48 60 and 72 hours during this period (vertical gray arrows). Incidence of vaginal spotting (pink) and frank menstruation (red) on each day are shown as bars across the x axis. Each bar represents one animal. Replacement of P at 12, 24 and 30 hours blocked frank menses. Replacement of P at 36 h blocked bleeding in all but one animal, at 48 h failed to block bleeding in three out of 4 animals, and P replacement later than 48 h after P withdrawal failed to block bleeding in all animals.

Figure 7 shows that when the P implants were replaced at 12, 24 and 30 h in a total of 10 animals, menses was completely inhibited in 9; one of those in which P was replaced at 24 h showed some spotting. Replacement at 36 and 40 h blocked frank menses in 5 out of 6 animals. Menstrual spotting occurred in the two animals where P was replaced at 40 h. Progesterone replacement at 48 h failed to block frank menses in 3 out of 4 animals, though menstruation was slightly attenuated with more days of spotting. P replacement at 60 and 72 h failed to block menses in all the animals. When menses was not blocked by adding back P, bleeding generally began on day 2 of the LFT as in control animals. Therefore, the first 36–40 hours after P withdrawal defines a critical period for frank menses induction since P replacement before but not after that period inhibited menses. This conclusion is based on the time of onset of frank menstrual bleeding on the perineum, not spotting, because the latter is a highly variable endpoint that can be affected by rate of seepage, strength of contractions and other factors. The menstrual pattern in pigtailed macaques that received the blank implants was identical to the pattern presented in Fig. 3.

### Effects of P replacement on MMPs

**Figure 8:**
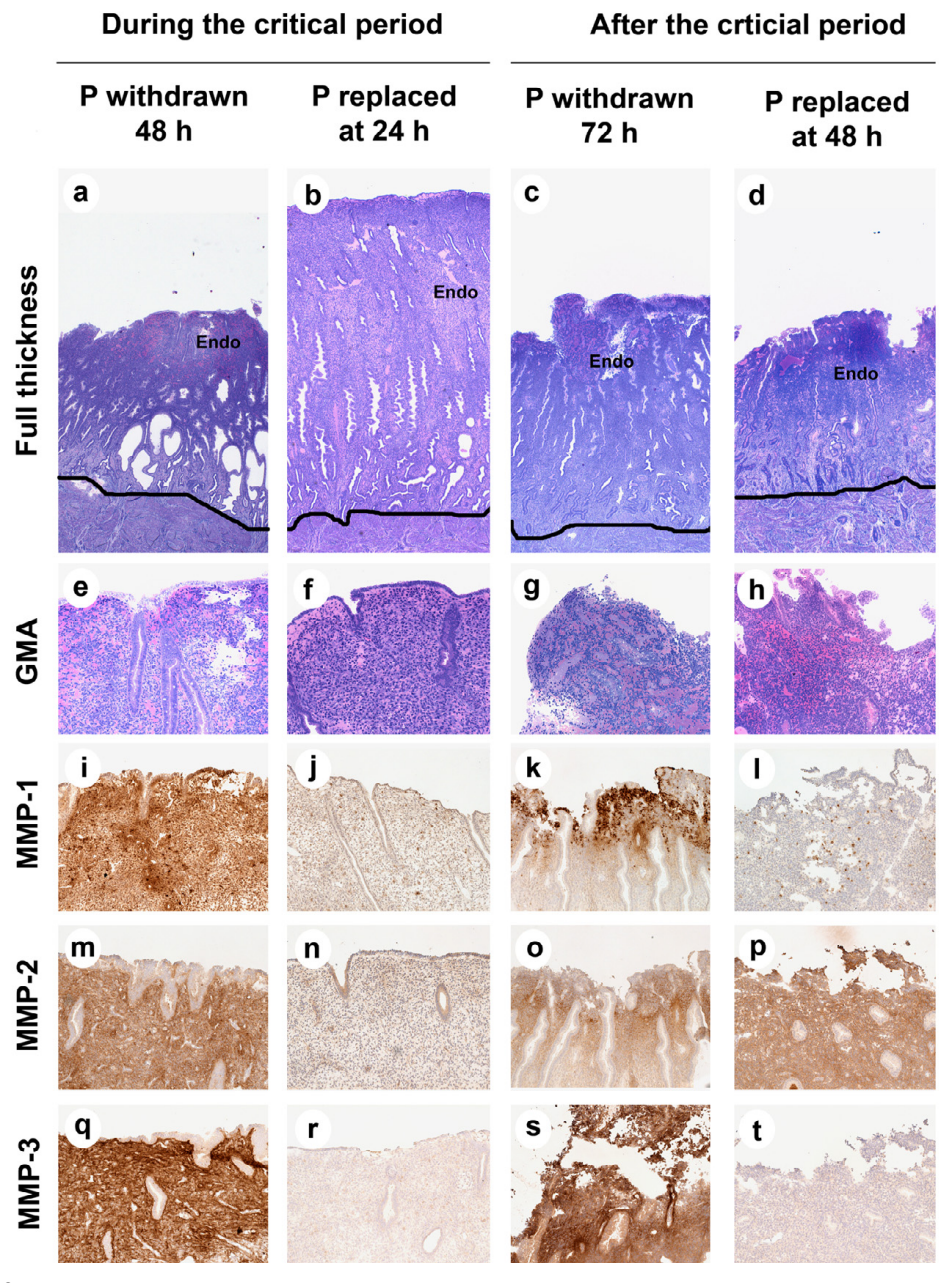
Effect of P replacement on endometrial histology and MMP ICC. The photomicrographs are arranged as a grid with each treatment in columns and histological endpoints in each row. a-h: GMA sections showing endometrial histology (a-d; Magnification 100× original magnification and e-h; functionalis zone, Original Magnification 25×). i-t: ICC for MMPs. Strong staining for MMP1, MMP2, and MMP-3 was observed at 48 and 72 h in control animals. Replacement of P during, but not after, the critical period blocked menstrual breakdown (compare b to d and f to h). Replacement of P at 24 hours blocked expression of all three MMPs (j, n, r), but P replacement at 48 hours blocked MMP-1 and MMP-3 but not MMP-2 (l,p,t).

Figure 8 shows that P replacement before, but not after, 48 h prevented the histological signs of menstrual breakdown. Strong immunocytochemical staining for MMP1, MMP2, and MMP-3 was evident in controls at 36, 48 and 72 h. P replacement during the critical period, which blocked menses, blocked expression of all three MMPs. However, P replacement after the critical period, which failed to suppress menses, suppressed MMP-1 and MMP-3 but not MMP2. Therefore MMP-1 and -3 may be more important for initiating menses than for sustaining menstrual breakdown, and MMP2 may be more important than MMP-1 and -3 in sustaining the menstrual breakdown process. We conclude that different MMPs vary in their sensitivity to P suppression and play different roles in menstrual breakdown.

### The two-step hypothesis of menstrual induction

Our studies support the view that menstrual induction occurs in two phases [19]. As we have shown, there is a first critical phase, followed by a second, in which menses becomes irreversible. But little is known about the underlying factors that mediate these steps toward inevitable breakdown. P normally suppresses the NFκB system in PR-positive, endometrial stromal cells in part by elevating its inhibitor, IκB, which keeps NFκB sequestered in the cytoplasm in an inactive state [23]. When P is withdrawn at the end of the cycle, intracellular IκB levels fall and NFκB translocates into the nucleus and activates transcription of various cytokine genes. These events occur first in PR-positive cells, and lead either directly or indirectly to an increase in cyclooxgenase-2 (COX-2), which results in increased synthesis of prostaglandins [24]. During the luteal phase, P increases the levels of the metabolic enzyme, prostaglandin dehydrogenase [25], which keeps prostaglandin levels low. When P levels decline, prostaglandin dehydrogenase activity decreases. In the premenstrual phase, the combined effect of the increase in COX-2 and the decrease in prostaglandin dehydrogenase leads to elevated prostaglandins, which are potent vasoconstrictors. Vasoconstriction has long been assumed to induce anoxic injury that plays a role in the menstrual cascade [26].

Activated NFκB also enhances the secretion of cytokines that affect vascular permeability, induce leucocyte invasion, and further stimulate the secretion of prostaglandins and endothelins. Many leucocytic cell types secrete MMPs and other lytic enzymes which facilitate tissue breakdown and bleeding [27]. Because endometrial leucocytes and macrophages are PR-negative, adding back P would not directly affect their synthetic activities. With time, endothelial cells, which are also PR-negative, initiate synthesis of endothelins, and the invading leucocytes contribute their lytic effects to the menstrual cascade. Finally, once elevated prostaglandins affect the myometrium and induce contractions strong enough to expel the menstrual slough, the menstrual process becomes irreversible.

The PR-positive perivascular cells that surround and support the spiral arterioles are, therefore, major players in the menstrual cascade. Two cytokines that are generated specifically within the perivascular stroma are IL-8 and MCP-1. These are among the cytokines that attract multiple leucocytic cell types into the endometrium. By utilizing an experimental model of P withdrawal in women, Critchley et al [24] demonstrated by northern analysis that IL-8 and COX-2 mRNA and protein were substantially upregulated by 48 hours after P withdrawal. Furthermore, IL-8 mRNA expression, as demonstrated by in situ hybridization, was primarily expressed by the perivascular cells in late secretory endometrium [28]. This locus of IL-8 expression facilitates the attraction and inward migration of leucocytes into the endometrium.

## Conclusion

**Figure 9:**
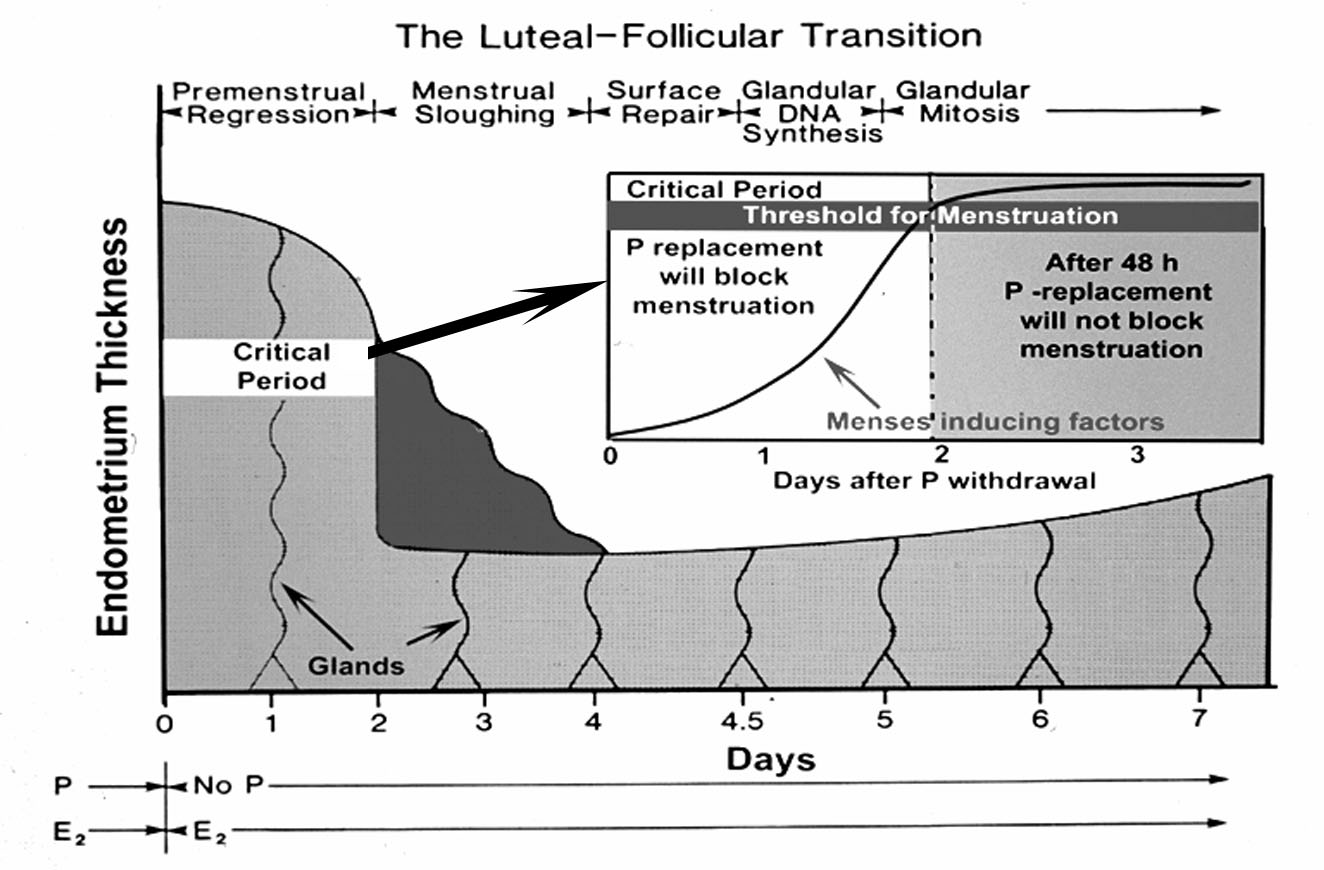
The Luteal-Follicular Transition. This diagram illustrates the various phases that follow P withdrawal in the primate endometrium, beginning with Premenstrual Regression, Menstrual Sloughing, Suface Repair, etc. The critical period is shown as a 2 day period during which menses-inducing factors build up to a threshold of inevitability.

As summarized in Figure 9, progesterone replacement during the first 36–48 hours of P withdrawal can suppress MMP expression and menstrual breakdown. After that critical period, menses becomes refractory to P add back. These results support the hypothesis originally proposed by Kelly et al [19] that menstrual induction consists of two separate phases. Our findings lay the groundwork for a much finer, time-based analysis of the cytokine-regulatory networks underlying the critical period. This sort of experimental analysis can be efficiently conducted in hormonally manipulated nonhuman primates, and a treasure trove of information awaits the use of modern methods of molecular biology to analyze such time-based data. Because disorders of uterine bleeding remain a major clinical problem and health burden, advances in our understanding of these processes have great potential to improve women's health.
